# Influences of tea consumption on self‐rated health and life satisfaction among older adults: Evidence from the CLHLS

**DOI:** 10.1002/pchj.807

**Published:** 2024-10-27

**Authors:** Min Zou, Changlong Sun, Mengxue Yang, Changjiang Li, Shuping Wang, Dewei Zheng, Jiali Wang, Lirong Yu, Lina Sun, Yanyu Wang, Huashuai Chen, Yi Zeng

**Affiliations:** ^1^ School of Psychology Shandong Second Medical University Weifang China; ^2^ School of Public Health, Cheeloo College of Medicine Shandong University Jinan China; ^3^ Shandong College of Economics and Business Weifang China; ^4^ School of Nursing Shandong Second Medical University Weifang China; ^5^ School of Anesthesiology Shandong Second Medical University Weifang China; ^6^ Department of Management Business School of Xiangtan University Xiangtan China; ^7^ Centre for the Study of Aging and Human Development and Geriatrics Division Medical School of Duke University Durham North Carolina USA; ^8^ Centre for Healthy Aging and Development Studies, National School of Development Peking University Beijing China

**Keywords:** older adults, self‐rated health, self‐rated life satisfaction, tea consumption

## Abstract

The benefits of tea consumption as a special diet for health and life satisfaction have attracted considerable attention; however, it is not clear whether the effect of tea consumption on self‐rated health (SRH) and self‐rated life satisfaction (SRL) is equal among all types of tea, and it is unclear whether these associations are impacted by gender and age in older adults. This study aimed to examine the associations between tea consumption, SRH and SRL in older adults and to explore the role of gender and age. Participants aged 65–105 (*N* = 78,345) were interviewed in the years 2002, 2005, 2008, 2011, 2014 and 2018 in the Chinese Longitudinal Healthy Longevity Study (CLHLS). Generalized estimation equations (GEE) with the identity link function were adopted to estimate the cross‐sectional associations of tea consumption with SRH and SRL. GEE with the logic link function were used to explore the longitudinal associations of tea consumption with SRH decline and SRL decline. Drinking tea at present, especially scented tea, was significantly associated with better SRH and SRL for older adults. Male participants benefited more from tea consumption than females, and the protective effect of green tea consumption on improving SRH and SRL in males was evident. Older adults aged 90–105 with current tea consumption daily had better SRH and reduced risk of SRL decline.

## INTRODUCTION

The world has seen a significant ageing trend since the 21st century. The life expectancy of older individuals has been rising year after year with the advances in medical care; however, majority of old people are in the ‘unhealthy’ or ‘inactive’ state in spite of longer living (Goroshko et al., 2021). The issue of healthy longevity is receiving considerable scholarly attention in such a context and the common goal of healthy ageing and active ageing promoted by the United Nations is to maintain activities and life satisfaction of older adults, in which it emphasizes the vital connection between activities and health (Walker, 2002). Not surprisingly, health and life satisfaction are core elements of healthy ageing and active ageing, and they are commonly measured by self‐rated questionnaires (Ghubach et al., 2010).

Self‐rated health (SRH) is the subjective evaluation and expectation of an individual on his or her health status, which is considered to be the most commonly used measure of an individual's overall health status and valuable indicator of current and future health status in National Health Interview Survey (Li et al., 2011). Self‐rated life satisfaction (SRL) is a subjective evaluation of the quality of life based on the standard set by individuals, which is an important psychological parameter to measure the life of people in a society (Wang et al., 1999). Briefly, SRH and SRL can assess the holistic health and life status of older adults expediently and comprehensively. In order to promote healthy and active ageing in older adults, researchers have been searching for optimal solutions from the perspective of prevention from ageing.

It is known that Japanese people have the longest life expectancy in the world mainly due to a low mortality rate from heart disease, which is considered to be attributable to their Japanese diet including tea drinking (Kuriyama et al., 2006; Momiyama et al., 2023; Tsugane, 2020). Tea as a special diet has attracted considerable attention since the 1960s. Extensive studies on association between tea and diseases (Chan et al., 2018; Gan et al., 2018; Kishimoto et al., 2020) have shown the beneficial effects of tea drinking in preventing diabetes and its complications (Meng et al., 2019), reducing the incidence of cardiovascular disease (Bahorun et al., 2012), preventing Alzheimer's diseases (Feng et al., 2015) and improving cognitive function (Feng et al., 2012).

Physical diseases are viewed as considerable factors of impairing an individual's SRH and SRL (Hou et al., 2007; Liu & Guo, 2008; Meng et al., 2014; Wu et al., 2013). Evidence had shown that drinking tea could be beneficial to the improvement of physical diseases, but the findings from various studies on association between SRH/SRL and tea consumption in older adults have been inconsistent. Some studies concluded that habitual tea consumption was positively associated with SRH and SRL in older adults (Pan et al., 2017; Qiu et al., 2012; Wang et al., 2021); conversely, others found that daily tea consumption was as effective as little or no tea consumption in promoting SRH and SRL (An et al., 2018; Shen et al., 2010). The reasons for these different findings were analysed. On the one hand, the effects of drinking tea on diseases and health could depend on tea consumption frequencies and types (Noguchi‐Shinohara et al., 2014). However, prior studies on association between tea consumption and SRH/SRL had not taken tea consumption frequencies and types into account. On the other hand, gender and age could be the vital variables in protective effect of tea consumption on quality of life and against diseases (Hou et al., 2007; Xu et al., 2018), but there are few studies on gender‐specific and age‐specific differences in association between tea consumption and SRH/SRL.

Therefore, this study aimed to examine the association between tea consumption (frequencies and types) and SRH/SRL among older adults and to delve into the differential effects of tea consumption (frequencies and types) on SRH/SRL in gender and age. Considering that previous studies were primarily cross‐sectional, our study had the advantage of examining the longitudinal association between tea consumption and SRH decline and SRL decline among older adults.

## METHODS

### Participants

The data used in this study were from the dataset of the Chinese Longitudinal Healthy Longevity Survey (CLHLS). The CLHLS aimed to examine the health determinants of older individuals, initiated in 1998 by the Center for Healthy Aging and Development Studies at Peking University's National School of Development. From 1998 to 2018, the CLHLS had been carried out eight waves (1998, 2000, 2002, 2005, 2008, 2011, 2014 and 2018) in about half of 23 randomly selected counties and urban areas in 31 provinces in China. Questionnaire data of the CLHLS were collected to provide extensive personal information of older participants including tea consumption, SRH, SRL, gender, age, region, rural/urban residence, education, marital status, co‐residence with children, family income, smoking, alcohol consumption and physical exercise.

Data of participants aged 65–105 for this study were from the dataset which was derived from six waves (2002, 2005, 2008, 2011, 2014 and 2018) of CLHLS, due to the information of survey only for participants aged over 80 years in 1998 and 2000. The dropout rate of participants in 2005, 2008, 2011, 2014 and 2018 was 49.1%, 52.2%, 52.2%, 37.9% and 16.3%, respectively. To compensate for attrition of participants due to death or loss in follow‐up wave and maintain study consistency, the CLHLS incorporated new participants who could mirror the characteristics of the departed. After removing those without information on SRH, SRL or tea consumption, 78,345 participants aged 64–105 were involved. Participant characteristics on SRH, SRL and tea consumption are reported in Table 1.

**TABLE 1:** Characteristics of the samples on SRH, SRL and tea consumption.

Characteristics	2002	2005	2008	2011	2014	2018
	*N* = 15,791	*N* = 15,354	*N* = 16,199	*N* = 9297	*N* = 6676	*N* = 15,028
Self‐reported health score, mean (*SD*)	2.60 (0.92)	2.57 (0.93)	2.57 (0.93)	2.64 (0.92)	2.64 (0.88)	2.57 (0.90)
Self‐rated life satisfaction score, mean (*SD*)	2.34 (0.83)	2.33 (0.83)	2.36 (0.82)	2.29 (0.82)	2.20 (0.79)	2.11 (0.80)
Poor self‐reported health, *n* (%)	2436 (15.4)	2321 (15.1)	2343 (14.5)	1523 (16.4)	968 (14.5)	1949 (13.0)
Poor self‐rated life satisfaction, *n* (%)	7031 (44.5)	6775 (44.1)	7734 (47.7)	4057 (43.6)	2519 (37.7)	5395 (35.9)
Frequencies of tea consumption at present						
Daily, *n* (%)	4711 (29.8)	4346 (28.3)	4900 (30.2)	2202 (23.7)	1345 (20.1)	2482 (16.5)
Occasionally, *n* (%)	2930 (18.6)	2895 (18.9)	2567 (15.8)	1528 (16.4)	994 (14.9)	1267 (8.4)
Rarely/never, *n* (%)	8150 (51.6)	8113 (52.8)	8732 (53.9)	5567 (59.9)	4336 (64.9)	11,279 (75.1)
Frequencies of tea consumption at around 60 years						
Daily, *n* (%)	4289 (27.5)	4287 (28.2)	4531 (28.0)	2610 (28.2)	1634 (24.7)	2486 (17.2)
Occasionally, *n* (%)	3188 (20.5)	3082 (20.2)	3197 (19.8)	1810 (19.6)	1256 (19.0)	1589 (11.0)
Rarely/never, *n* (%)	8103 (52.0)	7853 (51.6)	8427 (52.2)	4829 (52.2)	3712 (56.2)	10,380 (71.8)
Types of tea at present						
Green tea, *n* (%)	–	–	–	–	882 (15.4)	1863 (12.7)
Oolong/white tea, *n* (%)	–	–	–	–	96 (1.7)	218 (1.5)
Black/dark tea, *n* (%)	–	–	–	–	107 (1.9)	559 (3.8)
Scented tea, *n* (%)	–	–	–	–	320 (5.6)	697 (4.8)
Rarely/never, *n* (%)	–	–	–	–	4336 (75.5)	11,279 (77.2)
Types of tea at around age 60 years						
Green tea, *n* (%)	–	–	–	–	633 (13.0)	1668 (12.4)
Oolong/white/yellow tea, *n* (%)	–	–	–	–	105 (2.2)	220 (1.6)
Black/dark/compressed tea, *n* (%)	–	–	–	–	81 (1.7)	461 (3.4)
Scented tea, *n* (%)	–	–	–	–	325 (6.7)	672 (5.0)
Rarely/never, *n* (%)	–	–	–	–	3712 (76.4)	10,380 (77.5)
Covariates						
Age: 65–79, *n* (%)	4843 (30.7)	4955 (32.3)	4282 (26.4)	3135 (33.7)	2304 (34.5)	5179 (34.5)
80–89, *n* (%)	4237 (26.8)	3909 (25.5)	4275 (26.4)	2615 (28.1)	2152 (32.2)	3882 (25.8)
90–99, *n* (%)	3746 (23.7)	3952 (25.7)	4619 (28.5)	2401 (25.8)	1603 (24.0)	3440 (22.9)
100–105, *n* (%)	2965 (18.8)	2538 (16.5)	3023 (18.7)	1146 (12.3)	617 (9.2)	2527 (16.8)
Female, *n* (%)	9008 (57.0)	8711 (56.7)	9265 (57.2)	5037 (54.2)	3541 (53.0)	8412 (56.0)

All surveys were administered through in‐person interviews at the participants' residences, with each participant providing a signed informed consent form. This study adhered to the principles of the Declaration of Helsinki and was approved by the Institutional Review Board of Peking University.

### Measurements

SRH and SRL were assessed by the questions ‘How do you rate your health at present?’ and ‘How do you rate your life at present?’ The commonly used single‐item method was found to be reliable and valid (Cheung & Lucas, 2014; Macias et al., 2015). The responses included ‘1 = *very good*’, ‘2 = *good*’, ‘3 = *so‐so*’, ‘4 = *bad*’ and ‘5 = *very bad*’. The smaller the score, the better the status of SRH or SRL.

The SRH was dichotomized in a way such that subjects reporting ‘bad’ or ‘very bad’ were considered to have a poor SRH (assigning a value of 1) and subjects reporting ‘very good’, ‘good’ or ‘so‐so’ were categorized as having a good SRH (assigning a value of 0). So was SRL. SRH decline was defined when SRH was good in the current wave but poor in the follow‐up wave (Castellanos‐Perilla et al., 2020; Chang et al., 2024). So was the SRL decline.

The frequencies and types of tea consumption were assessed with information on tea drinking at present and at around 60 years old. Three choices of tea consumption frequencies were defined as ‘rarely/never’, ‘occasionally’ and ‘daily’, and four choices of tea consumption types for tea drinkers were defined as ‘green tea (unfermented tea)’, ‘oolong/white tea (semi‐fermented tea)’, ‘black/dark tea (fermented tea)’ and ‘scented tea’.

### Statistical analysis

Statistical analyses of the questionnaire data were performed using statistical software Stata 17.1. Generalized estimation equations (GEE) were adopted to explore the associations of tea consumption with SRH and SRL, respectively. We used the identity link function and reported the coefficients and 95% confidence interval (CI) obtained from the model estimated robust standard errors. Exchangeable correlation structure was used to account for subject level repeated measures. In order to delve into the longitudinal associations of tea consumption with SRH decline and SRL decline, respectively, we used the logit link function and reported the odds ratio (OR) and 95% CI obtained from the model estimated robust standard errors. Participants with good SRH/SRL (assigning a value of 0) in current wave were remained after removing those with poor SRH/SRL in this wave. There was SRH/SRL decline when participants with good SRH/SRL in current wave reported poor SRH/SRL (assigning a value of 1) in follow‐up wave (Castellanos‐Perilla et al., 2020; Chang et al., 2024). It represented reduced risk of SRH decline or SRL decline when a value of OR was smaller than 1. Participants in the reference group were those with good SRH/SRL across all the current and follow‐up waves.

Three models were used to portray the results. Model 1 was the base model unadjusted. Model 2 was adjusted for demographic variables including age, gender, region, rural/urban residence, education, marital status, co‐residence with children and family income. Model 3 was further adjusted for health‐related behaviour variables including smoking, alcohol consumption, physical exercise and dummies of waves based on model 2. In addition, stratified analyses by gender and age were conducted respectively based on model 3.

## RESULTS

### Cross‐sectional associations of tea consumption frequencies with SRH

The association between tea consumption frequencies and SRH in model 1 and model 2 showed significantly smaller SRH scores in participants drinking tea (occasionally and daily) at present or at around 60 years than in those who rarely drank tea (see Table 2). The regression coefficients were − 0.07 (95% CI = −0.09, −0.05) and −0.13 (95% CI = −0.15, −0.11) respectively in older adults drinking tea occasionally and daily at present, when demographic variables were adjusted in model 2. The association remained statistically significant even after further controlling for smoking, alcohol consumption, physical exercise and dummies of waves (see model 3, Table 2). However, the regression coefficients in people drinking tea at around 60 years were not statistically significant compared with nondrinkers, after additional adjustments for smoking, alcohol consumption, physical exercise and wave variables (see model 3, Table 2).

**TABLE 2:** Cross‐sectional associations of tea consumption frequencies with SRH and SRL.

		Association between tea consumption frequencies and SRH								Association between tea consumption frequencies and SRL						
		Tea consumption at around 60 years				Tea consumption at present				Tea consumption at around 60 years				Tea consumption at present		
		Rarely (*N* = 38,870)	Occasionally (*N* = 13,105)	Daily (*N* = 18,823)		Rarely (*N* = 41,153)	Occasionally (*N* = 11,461)	Daily (*N* = 19,080)		Rarely (*N* = 38,852)	Occasionally (*N* = 13,099)	Daily (*N* = 18,804)		Rarely (*N* = 41,129)	Occasionally (*N* = 11,452)	Daily (*N* = 19,074)
Mean (*SD*)		2.63 (0.92)	2.58 (0.91)	2.53 (0.92)		2.65 (0.92)	2.56 (0.89)	2.48 (0.90)		2.30 (0.82)	2.27 (0.81)	2.24 (0.82)		2.31 (0.83)	2.28 (0.80)	2.21 (0.81)
Coef. [95% CI]	Model 1	0.00	−0.04 [−0.06, −0.02]	−0.09 [−0.11, −0.08]	0.00		−0.09 [−0.11, −0.07]	−0.17 [−0.18, −0.15]	0.00		−0.03 [−0.04, −0.01]	−0.06 [−0.07, −0.04]	0.00		−0.02 [−0.04, 0.00]	−0.09 [−0.10, −0.08]
	Model 2	0.00	−0.02 [−0.04, 0.00]	−0.04 [−0.06, −0.03]	0.00		−0.07 [−0.09, −0.05]	−0.13 [−0.15, −0.11]	0.00		0.00 [−0.02, 0.01]	0.00 [−0.02, 0.01]	0.00		−0.01 [−0.03, 0.01]	−0.06 [−0.07, −0.04]
	Model 3	0.00	−0.01 [−0.03, 0.01]	−0.01 [−0.03, 0.01]	0.00		−0.05 [−0.07, −0.03]	−0.09 [−0.10, −0.07]	0.00		−0.02 [−0.04, 0.00]	−0.01 [−0.03, 0.00]	0.00		−0.03 [−0.04, −0.01]	−0.07 [−0.08, −0.05]
Stratified analyses of model 3																
Male	Mean (*SD*)	2.59 (0.91)	2.53 (0.90)	2.47 (0.91)		2.63 (0.92)	2.52 (0.89)	2.42 (0.89)		2.32 (0.82)	2.26 (0.81)	2.20 (0.81)		2.33 (0.83)	2.28 (0.81)	2.18 (0.80)
	Coef. [95% CI]	0.00	−0.03 [−0.06, 0.00]	−0.04 [−0.06, −0.01]	0.00		−0.08 [−0.11, −0.05]	−0.12 [−0.15, −0.10]	0.00		−0.05 [−0.07, −0.02]	−0.05 [−0.07, −0.02]	0.00		−0.04 [−0.07, −0.02]	−0.09 [−0.12, −0.07]
Female	Mean (*SD*)	2.65 (0.92)	2.62 (0.91)	2.61 (0.92)		2.67 (0.92)	2.60 (0.89)	2.57 (0.91)		2.29 (0.83)	2.28 (0.81)	2.29 (0.82)		2.30 (0.83)	2.28 (0.80)	2.25 (0.82)
	Coef. [95% CI]	0.00	0.00 [−0.02, 0.03]	0.01 [−0.01, 0.04]	0.00		−0.04 [−0.06, −0.01]	−0.05 [−0.08, −0.03]	0.00		0.00 [−0.03, 0.02]	0.02 [0.00, 0.05]	0.00		−0.02 [−0.04, 0.01]	−0.03 [−0.06, −0.01]
Aged 65–79	Mean (*SD*)	2.61 (0.93)	2.51 (0.91)	2.47 (0.91)		2.63 (0.93)	2.49 (0.89)	2.44 (0.90)		2.33 (0.83)	2.26 (0.82)	2.22 (0.81)		2.34 (0.83)	2.27 (0.81)	2.20 (0.80)
	Coef. [95% CI]	0.00	−0.05 [−0.08, −0.01]	−0.04 [−0.08, −0.01]	0.00		−0.08 [−0.12, −0.05]	−0.11 [−0.14, −0.08]	0.00		−0.06 [−0.09, −0.03]	−0.05 [−0.08, −0.03]	0.00		−0.05 [−0.08, −0.02]	−0.10 [−0.12, −0.07]
Aged 80–89	Mean (*SD*)	2.67 (0.92)	2.62 (0.91)	2.60 (0.92)		2.71 (0.93)	2.61 (0.89)	2.52 (0.91)		2.33 (0.84)	2.29 (0.80)	2.27 (0.83)		2.35 (0.85)	2.30 (0.80)	2.22 (0.81)
	Coef. [95% CI]	0.00	−0.01 [−0.05, 0.02]	0.01 [−0.03, 0.04]	0.00		−0.06 [−0.09, −0.02]	−0.10 [−0.14, −0.07]	0.00		−0.03 [−0.06, 0.00]	0.00 [−0.03, 0.03]	0.00		−0.04 [−0.07, 0.00]	−0.08 [−0.11, −0.05]
Aged 90–105	Mean (*SD*)	2.61 (0.90)	2.60 (0.90)	2.54 (0.91)		2.63 (0.91)	2.58 (0.88)	2.50 (0.90)		2.27 (0.81)	2.26 (0.81)	2.23 (0.82)		2.27 (0.81)	2.27 (0.81)	2.21 (0.82)
	Coef. [95% CI]	0.00	0.03 [0.00, 0.06]	0.00 [−0.03, 0.03]	0.00		−0.02 [−0.06, 0.01]	−0.05 [−0.08, −0.03]	0.00		0.01 [−0.01, 0.04]	0.02 [0.00, 0.05]	0.00		0.00 [−0.03, 0.03]	−0.02 [−0.05, 0.00]

*Note*: Dependent variable: self‐rated health score (SRH) and self‐rated life satisfaction score (SRL). Model 1, base model; model 2, adjusted for age, gender, region, rural/urban residence, education, marital status, co‐residence with children, and family income; model 3: adjusted for smoking, alcohol consumption, physical exercise, and dummies of waves based on model 2.

Abbreviation: CI, confidence interval.

Stratified analyses by gender showed that males and females had similar smaller SRH scores when drinking tea (occasionally and daily) at present, while for male participants drinking tea at around 60 years, but not females, the SRH score was significantly smaller.

Stratified analyses by age showed that the coefficients of SRH for participants aged 65–79 drinking tea occasionally and daily at present were − 0.08 (95% CI = −0.12, −0.05) and −0.11 (95% CI = −0.14, −0.08), respectively, while for those aged 65–79 drinking tea at around 60 years was −0.05 (95% CI = −0.08, −0.01) and −0.04 (95% CI = −0.08, −0.01), respectively. The coefficients of SRH for participants aged 80–89 drinking tea occasionally and daily at present were − 0.06 (95% CI = −0.09, −0.02) and −0.10 (95% CI = −0.14, −0.07), respectively, but the coefficients for people aged 80–89 drinking tea at around 60 years was not statistically significant compared with nondrinkers. Besides, it was found that older adults aged 90–105 drinking tea daily at present reported a smaller SRH score than those who rarely or occasionally drank tea at present.

### Cross‐sectional associations of tea consumption frequencies with SRL

The regression coefficients of SRL were − 0.02 (95% CI = −0.04, 0.00) and −0.09 (95% CI = −0.10, −0.08), respectively, in older adults drinking tea occasionally and daily at present in model 1, and the association remained statistically significant even after further controlling for demographic and health‐related behaviour variables (see model 3, Table 2). However, the coefficients of SRL for people drinking tea at around 60 years had no significant difference compared with nondrinkers, when covariates were adjusted (see model 3, Table 2).

Stratified analyses by gender showed that males had smaller SRL score when drinking tea (occasionally and daily) either at present or at around 60 years. However, females had smaller SRL score only when drinking tea daily at present.

Stratified analyses by age showed that coefficients of SRL in participants aged 65–79 drinking tea occasionally and daily at present were −0.05 (95% CI = −0.08, −0.02) and −0.10 (95% CI = −0.12, −0.07), respectively, while for those aged 65–79 drinking tea at around 60 years, the coefficients were − 0.06 (95% CI = −0.09, −0.03) and − 0.05 (95% CI = −0.08, −0.03), respectively. Meanwhile, coefficients of SRL in participants aged 80–89 drinking tea occasionally and daily at present were − 0.04 (95% CI = −0.07, 0.00) and −0.08 (95% CI = −0.11, −0.05), respectively, but those for people aged 80–89 drinking tea at around 60 years were not significant compared with nondrinkers. Associations between SRL and drinking tea at present or at around 60 years in participants aged 90–105 were not significant compared with nondrinkers.

### Cross‐sectional associations of tea consumption types with SRH

Based on GEE models with SRH score as the dependent variable, associations of tea consumption types with SRH are presented in Table 3.

**TABLE 3:** Cross‐sectional associations of tea consumption types with self‐reported health.

		Tea consumption at around 60 years						Tea consumption at present				
		No tea (*N* = 12,838)	Green tea (*N* = 2220)	Oolong/white tea (*N* = 313)	Black/dark tea (*n* = 518)	Scented tea (*N* = 955)		No tea (*N* = 14,162)	Green tea (*N* = 2667)	Oolong/white tea (*N* = 301)	Black/dark tea (*N* = 640)	Scented tea (*N* = 990)
Mean (*SD*)		2.62 (0.89)	2.54 (0.92)	2.61 (0.91)	2.52 (0.89)	2.44 (0.91)		2.63 (0.89)	2.50 (0.91)	2.52 (0.90)	2.45 (0.89)	2.47 (0.88)
Coef. [95% CI]	Model 1	0.00	−0.09 [−0.13, −0.05]	−0.02 [−0.12, 0.08]	−0.10 [−0.18, −0.02]	−0.19 [−0.25, −0.13]		0.00	−0.13 [−0.17, −0.09]	−0.11 [−0.21, −0.01]	−0.17 [−0.24, −0.10]	−0.16 [−0.22, −0.10]
	Model 2	0.00	−0.02 [−0.07, 0.02]	0.07 [−0.04, 0.17]	−0.03 [−0.11, 0.05]	−0.11 [−0.17, −0.05]		0.00	−0.07 [−0.11, −0.03]	−0.04 [−0.15, 0.07]	−0.10 [−0.17, −0.02]	−0.11 [−0.17, −0.05]
	Model 3	0.00	0.02 [−0.03, 0.06]	0.09 [−0.02, 0.19]	0.04 [−0.04, 0.12]	−0.05 [−0.12, 0.01]		0.00	−0.03 [−0.07, 0.01]	−0.03 [−0.13, 0.08]	−0.03 [−0.11, 0.04]	−0.05 [−0.11, 0.01]
Stratified analyses of model 3												
Male	Mean (*SD*)	2.59 (0.88)	2.47 (0.92)	2.49 (0.91)	2.49 (0.88)	2.42 (0.90)		2.61 (0.89)	2.44 (0.91)	2.44 (0.90)	2.43 (0.88)	2.45 (0.86)
	Coef. [95% CI]	0.00	−0.02 [−0.08, 0.04]	0.02 [−0.12, 0.16]	0.03 [−0.07, 0.14]	−0.04 [−0.12, 0.04]		0.00	−0.08 [−0.13, −0.02]	−0.07 [−0.20, 0.07]	−0.04 [−0.13, 0.06]	−0.06 [−0.13, 0.02]
Female	Mean (*SD*)	2.65 (0.90)	2.65 (0.91)	2.76 (0.90)	2.58 (0.91)	2.46 (0.92)		2.65 (0.90)	2.62 (0.91)	2.64 (0.88)	2.49 (0.91)	2.50 (0.92)
	Coef. [95% CI]	0.00	0.08 [0.00, 0.15]	0.17 [0.01, 0.33]	0.03 [−0.10, 0.16]	−0.07 [−0.17, 0.03]		0.00	0.05 [−0.01, 0.12]	0.02 [−0.15, 0.20]	−0.05 [−0.16, 0.07]	−0.06 [−0.16, 0.04]
Aged 65–79	Mean (*SD*)	2.61 (0.90)	2.52 (0.91)	2.53 (0.85)	2.50 (0.88)	2.44 (0.94)		2.61 (0.90)	2.49 (0.90)	2.40 (0.86)	2.47 (0.88)	2.50 (0.92)
	Coef. [95% CI]	0.00	0.04 [−0.03, 0.10]	−0.03 [−0.18, 0.11]	0.02 [−0.09, 0.13]	−0.05 [−0.15, 0.05]		0.00	0.00 [−0.06, 0.06]	−0.14 [−0.29, 0.01]	−0.01 [−0.11, 0.10]	−0.02 [−0.12, 0.07]
Aged 80–89	Mean (*SD*)	2.65 (0.89)	2.61 (0.96)	2.72 (0.91)	2.61 (0.89)	2.47 (0.89)		2.67 (0.89)	2.56 (0.95)	2.60 (0.83)	2.53 (0.92)	2.47 (0.86)
	Coef. [95% CI]	0.00	0.07 [−0.02, 0.16]	0.21 [0.00, 0.42]	0.13 [−0.03, 0.28]	−0.05 [−0.17, 0.07]		0.00	0.00 [−0.08, 0.08]	0.02 [−0.17, 0.21]	0.01 [−0.13, 0.16]	−0.03 [−0.14, 0.09]
Aged 90–105	Mean (*SD*)	2.62 (0.89)	2.49 (0.88)	2.66 (1.01)	2.45 (0.92)	2.40 (0.88)		2.63 (0.89)	2.47 (0.88)	2.65 (0.99)	2.34 (0.87)	2.42 (0.85)
	Coef. [95% CI]	0.00	−0.05 [−0.13, 0.03]	0.21 [−0.01, 0.43]	−0.01 [−0.18, 0.17]	−0.07 [−0.18, 0.04]		0.00	−0.08 [−0.16, −0.01]	0.15 [−0.08, 0.38]	−0.15 [−0.30, 0.00]	−0.13 [−0.24, −0.01]

*Note*: Dependent variable: self‐rated health score. Model 1, base model; model 2, adjusted for age, gender, region, rural/urban residence, education, marital status, co‐residence with children, and family income; model 3, adjusted for smoking, alcohol consumption, physical exercise, and dummies of waves based on model 2.

Abbreviation: CI, confidence interval.

Results in model 1 showed significant coefficients of SRH in participants drinking green tea or scented tea at around 60 years compared with participants who rarely drank tea. However, when demographic variables were adjusted in model 2, the coefficient of SRH only in people drinking scented tea was significant. When health‐related behaviour variables were adjusted in model 3, there was no significant difference between tea‐drinkers at around 60 years and nondrinkers.

The coefficients of SRH in people drinking green tea and scented tea at present were − 0.13 (95% CI = −0.17, −0.09) and − 0.16 (95% CI = −0.22, −0.10), respectively, in model 1, and the association remained statistically significant after controlling for demographic variables in model 2. However, when smoking, alcohol consumption and physical exercise were adjusted, the coefficient of SRH only in people drinking scented tea at present (Coef. = −0.05, 95% CI = −0.11, −0.01) was significant (see model 3, Table 3).

Stratified analyses by gender showed that males, but not females, had smaller SRH score when drinking green tea at present. Stratified analyses by age showed that the coefficients of SRH for participants aged 90–105 drinking green tea and scented tea at present were − 0.08 (95% CI = −0.16, −0.01) and − 0.13 (95% CI = −0.24, −0.01), but this association was not found in older adults aged 65–89.

### Cross‐sectional associations of tea consumption types with SRL

Based on GEE models with SRL score as the dependent variable, associations of tea consumption types with SRL are presented in Table 4.

**TABLE 4:** Cross‐sectional associations of tea consumption types with self‐rated life satisfaction.

		Tea consumption at around 60 years						Tea consumption at present				
		No tea (*n* = 12,840)	Green tea (*N* = 2217)	Oolong/white tea (*N* = 312)	Black/dark tea (*N* = 518)	Scented tea (*Nn* = 957)		No tea (*N* = 14,164)	Green tea (*N* = 2668)	Oolong/white tea (*N* = 300)	Black/dark tea (*N* = 640)	Scented tea (*N* = 989)
Mean (*SD*)		2.18 (0.80)	2.02 (0.79)	2.14 (0.80)	2.07 (0.80)	1.93 (0.78)		2.19 (0.80)	2.00 (0.79)	2.09 (0.81)	2.04 (0.78)	1.93 (0.77)
Coef. [95% CI]	Model 1	0.00	−0.16 [−0.20, −0.12]	−0.05 [−0.13, 0.04]	−0.11 [−0.18, −0.03]	−0.25 [−0.30, −0.20]		0.00	−0.18 [−0.21, −0.14]	−0.09 [−0.18, 0.00]	−0.14 [−0.20, −0.08]	−0.25 [−0.30, −0.20]
	Model 2	0.00	−0.09 [−0.12, −0.05]	0.02 [−0.07, 0.11]	−0.01 [−0.08, 0.07]	−0.18 [−0.23, −0.13]		0.00	−0.11 [−0.14, −0.07]	−0.04 [−0.14, 0.06]	−0.05 [−0.12, 0.01]	−0.22 [−0.27, −0.16]
	Model 3	0.00	−0.07 [−0.10, −0.03]	0.04 [−0.05, 0.13]	0.02 [−0.05, 0.10]	−0.15 [−0.20, −0.09]		0.00	−0.08 [−0.12, −0.05]	−0.02 [−0.12, 0.08]	−0.02 [−0.09, 0.04]	−0.19 [−0.24, −0.13]
Stratified analyses of model 3												
Male	Mean (*SD*)	2.20 (0.79)	2.00 (0.77)	2.05 (0.75)	2.07 (0.80)	1.97 (0.80)		2.19 (0.79)	1.99 (0.78)	2.09 (0.83)	2.06 (0.79)	1.96 (0.79)
	Coef. [95% CI]	0.00	−0.09 [−0.14, −0.04]	−0.04 [−0.16, 0.08]	0.02 [−0.08, 0.12]	−0.11 [−0.19, −0.04]		0.00	−0.10 [−0.14, −0.05]	0.00 [−0.13, 0.13]	0.01 [−0.08, 0.09]	−0.15 [−0.22, −0.08]
Female	Mean (*SD*)	2.17 (0.80)	2.05 (0.81)	2.26 (0.83)	2.07 (0.81)	1.86 (0.74)		2.18 (0.80)	2.02 (0.81)	2.09 (0.80)	2.01 (0.78)	1.88 (0.75)
	Coef. [95% CI]	0.00	−0.02 [−0.09, 0.04]	0.14 [0.00, 0.29]	0.01 [−0.11, 0.13]	−0.21 [−0.29, −0.13]		0.00	−0.05 [−0.11, 0.01]	−0.07 [−0.22, 0.09]	−0.07 [−0.18, 0.03]	−0.24 [−0.32, −0.16]
Aged 65–79	Mean (*SD*)	2.20 (0.81)	2.04 (0.80)	2.08 (0.78)	2.08 (0.77)	1.97 (0.79)		2.20 (0.81)	2.02 (0.81)	2.05 (0.80)	2.05 (0.76)	2.00 (0.81)
	Coef. [95% CI]	0.00	−0.04 [−0.10, 0.02]	−0.06 [−0.19, 0.06]	0.00 [−0.10, 0.10]	−0.13 [−0.22, −0.05]		0.00	−0.07 [−0.13, −0.02]	−0.12 [−0.26, 0.02]	−0.03 [−0.12, 0.06]	−0.14 [−0.22, −0.06]
Aged 80–89	Mean (*SD*)	2.18 (0.79)	2.00 (0.76)	2.27 (0.82)	2.17 (0.91)	1.94 (0.79)		2.20 (0.80)	1.99 (0.75)	2.15 (0.74)	2.11 (0.88)	1.87 (0.75)
	Coef. [95% CI]	0.00	−0.08 [−0.16, −0.01]	0.21 [0.02, 0.40]	0.14 [−0.02, 0.30]	−0.13 [−0.24, −0.03]		0.00	−0.09 [−0.16, −0.03]	0.04 [−0.14, 0.22]	0.02 [−0.12, 0.17]	−0.22 [−0.31, −0.12]
Aged 90–105	Mean (*SD*)	2.16 (0.79)	1.99 (0.78)	2.12 (0.81)	1.95 (0.72)	1.87 (0.76)		2.16 (0.78)	1.97 (0.79)	2.12 (0.89)	1.95 (0.71)	1.88 (0.75)
	Coef. [95% CI]	0.00	−0.09 [−0.16, −0.01]	0.08 [−0.11, 0.27]	−0.09 [−0.24, 0.06]	−0.17 [−0.27, −0.08]		0.00	−0.08 [−0.15, −0.01]	0.08 [−0.13, 0.30]	−0.07 [−0.20, 0.06]	−0.22 [−0.31, −0.12]

*Note*: Dependent variable: self‐rated life satisfaction score. Model 1, base model; model 2, adjusted for age, gender, region, rural/urban residence, education, marital status, co‐residence with children, and family income; model 3, adjusted for smoking, alcohol consumption, physical exercise, and dummies of waves based on model 2.

Abbreviation: CI, confidence interval.

Results in model 1 showed significant smaller SRL scores in participants drinking green tea or scented tea either at present or at around 60 years compared with participants who rarely drank tea. The regression coefficients of SRL in people drinking green tea and scented tea at present were − 0.11 (95% CI = −0.14, −0.07) and −0.22 (95% CI = −0.27, −0.16), respectively, when demographic variables were adjusted, while those in people drinking green tea and scented tea at around 60 years were −0.09 (95% CI = −0.12, −0.05) and − 0.18 (95% CI = −0.23, −0.13), respectively (see model 2, Table 4). The smaller SRL scores remained statistically significant for participants drinking green tea or scented tea either at present or at around 60 years after controlling for smoking, alcohol consumption and physical exercise (see model 3, Table 4).

Stratified analyses by gender showed that males had smaller SRL score when drinking green tea or scented tea either at present (Coef. = −0.10, 95% CI = −0.14, −0.05; Coef. = −0.15, 95% CI = −0.22, −0.08) or at around 60 years (Coef. = −0.09, 95% CI = −0.14, −0.04; Coef. = −0.11, 95% CI = −0.19, −0.04), while females had smaller SRL score when drinking scented tea either at present (Coef. = −0.24, 95% CI = −0.32, −0.16) or at around 60 years (Coef. = −0.21, 95% CI = −0.29, −0.13). Stratified analyses by age showed smaller SRL scores for older adults at each stages drinking green tea or scented tea at present or at around 60 years than those drinking other types of tea (see Table 4).

### Longitudinal associations of tea consumption frequencies with SRH decline and SRL decline

Based on GEE models with SRH decline and SRL decline across waves (ref. = 0) as the dependent variables, respectively, associations of tea consumption frequencies with SRH decline and SRL decline were explored, and OR and 95% CI with covariates adjusted are displayed in Table 5.

**TABLE 5:** Longitudinal associations of tea consumption frequencies with SRH decline and SRL decline.

	Association between tea consumption frequencies and SRH decline						Association between tea consumption frequencies and SRL decline					
	Tea consumption at around 60 years			Tea consumption at present			Tea consumption at around 60 years			Tea consumption at present		
	Rarely (*N* = 13,707)	Occasionally (*N* = 5398)	Daily (*N* = 8123)	Rarely (*N* = 14,024)	Occasionally (*N* = 4871)	Daily (*N* = 8484)	Rarely (*N* = 13,707)	Occasionally (*N* = 5398)	Daily (*N* = 8123)	Rarely (*N* = 14,024)	Occasionally (*N* = 4871)	Daily (*N* = 8484)
Sample [*n* (%)]	2005 (14.6)	766 (14.2)	1054 (13.0)	2028 (14.5)	692 (14.2)	1127 (13.3)	3383 (37.2)	1271 (34.2)	1880 (33.2)	3465 (37.3)	1177 (35.5)	1928 (32.4)
OR [95% CI]												
Model 1	1.00	0.97 (0.89, 1.06)	0.88 (0.81, 0.95)	1.00	0.98 (0.90, 1.08)	0.91 (0.84, 0.99)	1.00	0.89 (0.82, 0.96)	0.85 (0.79, 0.91)	1.00	0.92 (0.85, 1.00)	0.81 (0.76, 0.87)
Model 2	1.00	1.02 (0.93, 1.12)	0.98 (0.90, 1.07)	1.00	1.02 (0.93, 1.13)	1.00 (0.92, 1.09)	1.00	0.95 (0.87, 1.04)	0.98 (0.91, 1.06)	1.00	0.94 (0.86, 1.02)	0.90 (0.84, 0.97)
Model 3	1.00	1.02 (0.93, 1.12)	0.99 (0.90, 1.08)	1.00	1.02 (0.92, 1.12)	1.01 (0.92, 1.10)	1.00	0.94 (0.86, 1.02)	0.97 (0.90, 1.05)	1.00	0.91 (0.83, 0.99)	0.87 (0.81, 0.94)
*Stratified analyses of model 3*												
Male												
*n* (%)	707 (13.3)	372 (13.6)	593 (12.0)	718 (13.3)	305 (12.6)	656 (12.5)	1219 (36.0)	620 (33.6)	1120 (32.0)	1257 (36.6)	552 (34.2)	1164 (31.3)
OR [95% CI]	1.00	1.10 (0.96, 1.27)	1.00 (0.88, 1.14)	1.00	1.02 (0.88, 1.18)	1.04 (0.92, 1.18)	1.00	0.96 (0.85, 1.09)	0.94 (0.84, 1.05)	1.00	0.90 (0.79, 1.03)	0.84 (0.75, 0.93)
Female												
*n* (%)	1298 (15.5)	394 (14.8)	461 (14.6)	1310 (15.2)	387 (15.8)	471 (14.6)	2164 (37.9)	651 (34.7)	760 (35.3)	2208 (37.6)	625 (36.8)	764 (34.2)
OR [95% CI]	1.00	0.96 (0.84, 1.09)	0.99 (0.88, 1.12)	1.00	1.02 (0.90, 1.16)	0.98 (0.87, 1.11)	1.00	0.91 (0.81, 1.03)	1.01 (0.90, 1.13)	1.00	0.91 (0.81, 1.03)	0.92 (0.82, 1.03)
Aged 65–79												
*n* (%)	864 (15.1)	332 (13.9)	525 (12.3)	872 (14.9)	296 (13.3)	556 (12.8)	1338 (35.6)	546 (33.6)	943 (32.2)	1369 (35.8)	510 (34.0)	952 (31.7)
OR [95% CI]	1.00	0.99 (0.86, 1.14)	0.92 (0.81, 1.04)	1.00	0.93 (0.81, 1.08)	0.96 (0.85, 1.09)	1.00	0.98 (0.86, 1.11)	0.97 (0.86, 1.08)	1.00	0.91 (0.80, 1.04)	0.89 (0.80, 0.99)
Aged 80–89												
*n* (%)	628 (15.2)	262 (15.0)	343 (15.2)	633 (15.1)	246 (16.2)	364 (14.6)	1018 (35.5)	401 (32.7)	535 (33.5)	1024 (35.5)	373 (35.4)	571 (32.0)
OR [95% CI]	1.00	1.00 (0.85, 1.19)	1.13 (0.97, 1.33)	1.00	1.12 (0.94, 1.32)	1.06 (0.91, 1.24)	1.00	0.93 (0.80, 1.08)	1.07 (0.92, 1.23)	1.00	0.97 (0.83, 1.14)	0.92 (0.80, 1.05)
Aged 90–105												
*n* (%)	513 (13.3)	172 (13.6)	186 (11.6)	523 (13.1)	150 (13.4)	207 (12.4)	1027 (41.5)	324 (37.4)	402 (35.7)	1072 (41.4)	294 (38.6)	405 (34.6)
OR [95% CI]	1.00	1.11 (0.91, 1.36)	0.92 (0.76, 1.12)	1.00	1.06 (0.86, 1.31)	1.03 (0.85, 1.24)	1.00	0.88 (0.74, 1.04)	0.87 (0.74, 1.02)	1.00	0.86 (0.72, 1.02)	0.80 (0.68, 0.94)

*Note*: Dependent variable: self‐rated health decline across waves (ref. = 0) and self‐rated life satisfaction decline across waves (ref. = 0). Model 1, base model; model 2, adjusted for age, gender, region, rural/urban residence, education, marital status, co‐residence with children, and family income; model 3, adjusted for smoking, alcohol consumption, physical exercise, and dummies of waves based on model 2.

Abbreviations: CI, confidence interval; OR, odds ratio.

Results in model 1 showed a significantly reduced risk of SRH decline in participants drinking tea daily either at present or at around 60 years, but there was no significant association between tea consumption frequencies and reduced SRH decline risk after controlling for covariates, as shown in model 2 and model 3 (see Table 5). Besides, there was no significant association of tea consumption frequencies and reduced risk of SRH decline, and there was no difference in gender‐specific and age‐specific groups.

Results in Table 5 showed a significant reduction of SRL decline risk in participants drinking tea either at present or at around 60 years, compared with those who rarely drank tea in model 1. However, the reduced risk remained statistically significant only for participants drinking tea at present after controlling for demographic and health‐related behaviour variables. The SRL decline risk in people drinking tea occasionally and daily at present reduced to 0.91 (95% CI = 0.83, 0.99) and 0.87 (95% CI = 0.81, 0.94), respectively.

It was found from stratified analyses that male participants who drank tea daily at present were significantly less likely to suffer SRL decline (OR = 0.84, 95% CI = 0.75, 0.93) than those who rarely drank tea at present, but this association was not found in females (see Table 5). Besides, stratified analyses by age showed that risk of SRL decline in older adults aged 90–105 drinking tea daily at present was reduced significantly (OR = 0.80, 95% CI = 0.68, 0.94).

## DISCUSSION

It was found from the cross‐sectional analysis of older adults that drinking tea at present, especially scented tea, was significantly associated with better SRH and SRL, while the longitudinal analysis in this study showed the benefits of drinking tea at present for SRL. Furthermore, male participants benefited more from tea consumption than females, and the protective effect of green tea consumption on improving SRH and SRL in males was evident. Older adults aged 90–105 drinking tea daily at present had better SRH and reduced risk of SRL decline.

As many chronic diseases and disabilities can be influenced by diet and socioeconomic factors, there is still a high potential for unmeasured confounding on health and tea could be one of these factors. Most importantly and strikingly, this may be the first study to find that tea consumption at present predicted risk reduction of SRL decline in older adults, which confirmed the health benefits of tea drinking in older adults. Our finding is in consensus to an ageing health study in Hong Kong (Ho, 2021), which found that guiding older adults to establish a healthy tea drinking habit could boost their psychological well‐being. It was thought by neurobiologists that human emotions, including life satisfaction, are functions produced by the brain and that dopamine plays a crucial role in transmitting the ‘feel good’ message (Singh et al., 2022). Meanwhile, it was shown in various studies that theanine contained in tea could increase dopamine activity, and caffeine included in tea could increase the availability of dopamine receptors (Wang et al., 2022; Volkow et al., 2015), which could account for the enhanced life satisfaction in older adults who drink tea. In addition, this result of the association between drinking tea and better SRL may be related to health beliefs or other complex factors, which deserved to delve into.

The current study found that the effect of drinking green tea on SRH and SRL was more evident in men than women, which could be interpreted by the fact that men's bioavailability and metabolism to antioxidant nutrients such as tea polyphenols are different from women (Marino et al., 2011). Evidence from previous studies had indicated that the protective effect of green tea varied by gender, representing male‐specific significant association between green tea consumption and reduced disease risk such as mild cognitive impairment and Alzheimer's disease (Altmann et al., 2014; Xing et al., 2015; Xu et al., 2018), which may be related to SRH and SRL for older adults. In addition, the finding of more benefits from tea in males could be explained by the social factors in tea drinking activities. Since it has been a tradition for men drinking tea with friends or others in China, not alone, it could provide more opportunities to communicate with each other in such activities and promote SRH and SRL in older adults by increasing social interactions (Hoshi et al., 2020). Undoubtedly, this finding prompts us to independently consider protective and risk factors in males and females when explaining gender differences in the effects of tea consumption on SRH and SRL.

Various experimental studies had attempted to identify the mechanism responsible for the unique health‐promoting effects of green and scented tea compared with other types of tea. It was found that green tea contained the widest variety of catechins and their derivatives (Gao et al., 2016; Tang et al., 2019), while scented tea had not only the properties of green tea, but also the nutrients unique to flowers, which include protein and amino acids, with the peculiar flavour; thus, it set sensory and psychological pleasure and could greatly promote SRH and SRL of older adults (Guerdoux‐Ninot et al., 2016; Li et al., 2019; Wang, 2022). This explanation seems reasonable for the current finding that drinking scented tea in older adults was significantly associated with better SRH and SRL. However, some studies indicated that beneficial effect of tea consumption on health could be attributed to green tea (Goto et al., 2014). In addition, evidence from other studies indicated that protective effect of tea consumption on health was not limited to particular type of tea (Arab et al., 2009; Feng et al., 2010). Therefore, the underlying mechanism linking tea types to SRH and SRL is unclear, and it is necessary to elucidate the mechanism through further experimental studies.

It was found in this study that current tea consumption daily was significantly associated with better SRH and reduced risk of SRL decline in older adults aged 90–105, in accord with the finding from prior studies (An et al., 2018; Feng et al., 2012). It was possible that long‐term repeated tea consumption may result in lasting structural and functional changes in the brain of tea consumers, conferring protection against the development of poor health (Feng et al., 2012; Feng et al., 2016). Besides, evidence from prior studies had implied regular intake of tea compounds may activate expression of gene associated with longevity, which in turn may positively affect health status in the oldest old population (Zeng et al., 2015).

### Limitations

Specifically, we did not examine the psycho‐social aspects of tea consumption, whereby the association of tea consumption and SRH and SRL in older adults may be more in‐depth. On the one hand, this survey is unable to demonstrate the actual reasons for selecting various types of tea in older adults, no adjusting for the selection bias of participants. On the other hand, this survey is unable to examine whether participants often drank tea alone or with others. Due to social function of tea drinking with others, it is suggested that follow‐up studies should integrate both biological and psycho‐social aspects to study the mechanisms of tea consumption on SRH and SRL of older adults.

## CONFLICT OF INTEREST STATEMENT

The authors declare no conflicts of interest.

## ETHICS STATEMENT

This study was conducted in accordance with the Declaration of Helsinki and approved by the Institutional Review Board of Peking University (Approval No. IRB00001052‐13074). All participants were informed of the purpose and their rights as research participants, and written informed consent was obtained.
